# Learning from the nurses and the paramedics: the experience of a Kenyan medical officer intern-a call for research

**Published:** 2012-06-21

**Authors:** Aruyaru Stanley Mwenda

**Affiliations:** 1 Consolata Hospital, Nyeri, Kenya

**Keywords:** Internship, medical Education, skills, human resource, healthcare

## Abstract

In the Kenyan medical educations system, one has to go through one year of internship after graduating from medical school in order to be licensed to practise medicine. This internship period is laden with work to the extent of overwhelming and stressing the medical officer interns. Irrespective of what competence interns come with into the field there is still a lot they have to learn from the nurses and the paramedics. Most of the learning takes place during the acute care settings when the intern is on call and is from the nurses. The paramedics most helpful to the intern are the theater assistants who teach interns how to use the various surgical instruments and sometimes direct during minor operations.

## Background

The Kenyan medical system borrows heavily from the Pre-Registration House officer (PRHO) British medical system. In such a system, upon graduation from medical school, one is expected to complete a year of internship through the main departments of medicine, pediatrics, obstetrics and gynecology and surgery before they are licensed to join the league of medical practitioners either as government employees, private sector employees or private practitioners. In Kenya, universities offering undergraduate training in medicine include the university of Nairobi and Moi University. Kenyatta, Egerton and Maseno universities are the new entrants into this field [1] and as such up to last year only Moi University and University of Nairobi graduates were included in the ministry of medical services allocation of spaces for medical officer internship. The spirit of the internship program is that the newly graduated doctor should be able to learn new skills, apply the knowledge acquired in medical school and get initiation and mentorship into the field of medicine [2–5]. To achieve this, the young doctor is expected to work under supervision by seniors who are expected to mentor and assist the young doctor in various domains of patient management [4, 5].

As a medical officer intern, one is expected to admit patients, which entails history taking, physical examination and preliminary laboratory and radiological investigations, and do appropriate treatment plan, review patients after various investigations and modify the initiated management plan as may be necessary. These responsibilities cut across the departments. The medical officer intern is the house officer, she/he is supposed to know all the patients in the ward and their progress and is supposed to carry out all the bedside procedures like intravenous cannulation, drawing of blood samples, lumbar puncture and collection of other samples that might be needed for patient work up. The intern also does daily (or more frequent if necessary) rounds and reviews all the patients, writing discharge summaries for those fit to be discharged. When rotating in surgery, besides the above engagements, the medical officer intern is expected to work up the patients for theater (by ordering specific pre-operative investigations, obtaining consents and giving relevant pre-operative instructions) and be in theater to assist the surgeon or the senior medical officer and learn the various procedures.

In obstetrics and gynecology, it is the medical officer intern to do manual vacuum aspiration or dilatation and curettage for incomplete abortion, manual removal of placentae and review cases of difficult labor which the midwives may not feel comfortable handling. This is without mentioning the caesarean sections (CS) for the various indications. Apart from during the major rounds and operation days, the gynecologist may be rarely available during the other times and again the medical officer intern finds him/herself as the center around which everything revolves.

In pediatrics, it's the medical officer intern to resuscitate the cases of birth asphyxia, dehydration and coma. These may not be easy cases to the average medical officer intern especially in the early days of internship [2, 6] but the practice in Kenya is such that being unable to manage such, or say losing a baby to dehydration or shock would attract the wrath of having a few more weeks in the department. Findings of seniors reprimanding medical interns when consulted for ‘simple’ cases have been reported elsewhere in the literature [4, 5]. It is also established that there is a deficit in delineating between genuine medical interns limits and deficiency in patients care [5]. This Kenyan system, as noted above, is reflective of the PRHO British system which has been described as having a military hierarchy [5]. Such hierarchy and the fear of reprisals may often deter an intern from frequently consulting their seniors on duty especially when dealing with apparently ordinary cases.

The internship program is also such that the interns have to take shifts (calls) during nights and weekends. During the call time, one has to review all new admissions and respond to emergencies. Acute settings /crises, besides being life threatening to the patient, are often stressful and could even be life threatening to the young doctor too [4]. Depending on the size and business of a hospital, you may be expected to cover your department or the whole hospital when on call. In the smaller hospitals where you take calls across the whole hospital, you are expected to see patients with any imaginable condition whether medical or not, as long as they are in the hospital! It may just be the condition you have never met in medical school (‘unlike musicians who can play the same piece repeatedly.the practice of medical learners hinges on chance exposure to diseases’) [7]. It may be the condition you always never grasped in medical school [2, 8]. Sometimes the patient may not be as critical as may demand consulting the immediate superior (second on call). In cases where you are seeing patients from other departments when on call, it may be difficult due to fatigue and business to go back to the books and revise. As such wrong management may be carried forward until the intern is in the concerned department before discovering that he/she has been wrongfully prescribing, for example.

Medicine is such a diverse field and it would be unrealistic for one to expect you to know everything. That is why there is continuous medical education (CME) and a need for interns to update themselves from time to time [6]. But even with the latest information with you, it often occurs to you at the most critical moment that you need the technical knowhow besides textbook medicine [2, 5]. You need to administer a drug, or insert a cannula or a tube or do some other procedure. It is assumed that you have been taught all this in medical school. But as Greenburg et al conclude, it remains difficult to predict the performance of an intern based on their performance in medical school [8]. In some cases, the presentation of the patient fails to mirror what is in the book and the intern has to just assume a diagnosis for the sake of completion of the clerkship and justification of the management plan instituted [2]. In other cases, it's the outright knowledge deficit that becomes the stumbling block between a clinical presentation (when well elicited) and the correct diagnosis [2, 6]. In such scenarios, the senior should be able to direct the intern and take such an opportunity to teach them what is right to do. Against this background, it is worth noting that increased workload increases the risks of patient mortality [6] and exposure to occupational injuries among the interns [9].

The transition from medical school into the field is a period characterized by anxiety, stress and a multiple other challenges [2–5]. Much of this stress emanates from the work load, work duration and the new responsibilities [2–5]. The workload is sometimes so overwhelming that the stress takes the pathologic dimension of anxiety and depression which manifest as weight loss and/or insomnia [3]. Amidst this stress upon joining the field, various coping mechanisms by the medical officer interns have been cited [3]. Sadly such coping mechanisms sometimes have included substance abuse [3]. To the author's knowledge, no literature has analyzed any deficits that the graduating Kenyan doctor carries to the field and thus no one can blame the senior doctors and specialists of neglecting the intern when it comes to teaching simple practical aspects. This assumption goes on at the cost of quality patient care and optimal training for the intern. Goldacre and colleagues found out that besides variations among medical schools in terms of their preparation of medical students for the field, few of the young doctors under the PRHO system felt well prepared for their jobs [10].

Sometimes it may be difficult to get the senior doctor around the hospital especially outside the working hours. But there is always a nurse, often a paramedic wherever the intern is in the hospital. Tallentire and colleagues argue that a junior doctor's decisions in an acute care setting don not occur in isolation; instead they are influenced by his/her interactions with other members of the health care system and additional external artefacts [5]. These nurses and paramedics have a wealth of experience. As such, experienced nurses in rural Africa have been noted to perform procedures and other duties that are usually a preserve of specialists in urban areas and the western world [11]. With their wealth of experience irrespective of academic qualifications, these health care staff play a significant role in guiding the intern through especially when it comes to the technical know-how in the various spheres of internship. No study, to the best of the author's knowledge, has been done to find out the challenges the Kenyan medical officer interns face during their internship, neither is there data in the literature underscoring the crucial role of the nurses and the paramedics in the environment around the Kenyan medical officer intern. In this paper, the author analyses his experience during the period of internship.

### About Consolata hospital Nyeri

Consolata hospital Nyeri is a level IV catholic hospital currently with a work force that includes 55 nurses and 25 paramedics (laboratory technicians and technologists, pharmacy technicians, physiotherapists, radiographers and theater assistants). It is an internship center for both medical officer interns and clinical officer interns. It offers both in-patient and out-patient clinical services. The in-patient section is divided into four main departments: medicine, surgery, maternity (obstetrics and gynecology) and pediatrics.

During internship, one rotates through the four departments over a period of 12 months. The medical officer interns take weekday calls once every four days and a weekend call once a month. When on call, an intern is in charge of the whole hospital and reviews admissions, any patients that become unstable in the ward and does any surgeries either alone or with the assistance from a medical officer (second on call) or a consultant (third on call) depending on the complexity of the surgery. Outside the working hours, the hospital is literally under the control of nurses who should alert the intern on call in case of any patients that may need urgent review or for consultations where necessary. The paramedics offer axillary support including carrying out investigations requested by the doctors and helping in other ways in the patient management depending on their training and job description. It is a norm that the nurses cannot call the second or third on call without first calling the intern. As such, the intern sees each and every patient requiring review and makes the decision to consult the seniors (second and third on call). The author was placed (in turns) in the departments of pediatrics, obstetrics and gynecology, medicine and surgery.

## Learning themes

### Orientation into the new environment and responsibilities

It was a huge challenge settling into the new environment, with the new status and a huge responsibility. I found out that sometimes the practice in this hospital is different from what is learnt in books and practiced in the teaching hospital where I trained. To learn these I depended heavily on the nurses to know what they usually did under such circumstances. I realized right from the first day that the orientation that I was given by the hospital administrator was not the most important, it was the nurses’ direction for example in terms of what drugs they had in the pharmacy or what was the norm given a certain condition: ‘Doctor, that drug is not in our pharmacy but I usually see them given these tablets twice a day’, one nurse told me when I had prescribed a certain medication to a newly admitted patient. Then I learnt to enquire from the nurses more and more before I made other prescriptions only to be called to revise the treatment sheet. With time I was able to know which drugs were mostly out of stock and where the patients could be advised to purchase them. I also found out that at times the nurses would, based on their experience, disagree with part of my management and I had to follow their advice: ‘I am not going to give this patient this drip of 10% dextrose you have prescribed because I know he will definitely get hyperglycemia and I will be running around looking for the hospital phone to call you. So you can just go ahead and prescribe but for me I will not give it.’ After a while I thought against my initial plan and adjusted the treatment sheet.

### Learning during acute care settings and while on call

This was usually the most challenging time. I would be called to the out- patient casualty for example for a patient who had been involved in a road traffic accident. Such situations were overwhelming in the initial days and everything I had learned in medical school apparently would escape and sometimes I would be left staring at the patient after the initial initiation of fluids and some analgesics. Often I would call the second-on-call and get some instructions on the phone on what to do and what medications to prescribe. But even after prescribing and thinking that everything was alright, sometimes the nurse wound recommend another drug, or another investigation. Then I would be so amused at my oversight of certain common things because I had been carried away by the magnitude of the case.

In other instances the experienced nurse with the patient would have already initiated appropriate management by the time I arrived. I found certain cases not to be as serious as the nurse calling me had made it sound. But in certain cases where I needed to consult, or I was not 100 percent sure the nurse would sometimes be fully vindicated after my consultation. While doing my weekend call just before I joined obstetrics and gynecology, I was called for a patient in labor. She had been assessed by the midwife. The midwife informed me there was meconium stained liquor grade III but after assessment I thought it was grade II and directed close monitoring of labor. But the midwife was convinced there was fetal distress and the patient had to undergo a CS: “Sister get me that catheter and the blade and get the theater people on the phone, tell them to prepare for an emergency CS we have a fetal distress”, there was no convincing her otherwise. Intra-operatively there was a cord around the neck and the baby was delivered with mild birth asphyxia.

### Learning Practical skills

### Theater

The main learning area under this theme was theater. First of all, I learnt most of the instruments names and uses from the theater assistants and scrub nurses. Apart from a CS set, most of the other instruments were unfamiliar to me. I had heard the various types of sutures and even used them while assisting in theater as an undergraduate. But I knew little about which suture to use for which structure. The nurses would set the sutures ready and give me say vicryl number 1 for repairing the uterus. Some day when I got some serious bleeding while doing a CS I continued using vicryl number 1 but I could not achieve hemostasis. Then my scrub nurse just asked to be given vicryl 2/0. I thought she was getting the suture ready for the peritoneum. Then she handed me the suture saying: “This is what will save us here, doctor”. True to her advice we had hemostasis within no time.

Even doing real surgical procedures I also heavily relied on theater assistants and scrub nurses. My first dilatation and curetage (D&C) for an incomplete abortion was virtually done by my assistant. Giving me the in-and-out catheter to empty the bladder when I wanted to go directly to curettage, reminding me to do a bimanual pelvic examination when I asked him for the curette and then giving me the uterine sound and showing me how to use it when I had no idea what it was. I always felt safe when he was the one scrubbing with me during subsequent days.

### Wards

Compared to theater, there was not as much to learn from the wards in terms of practical skills. The aspect where much learning took place was in terms of which laboratory samples to take for which test and in what kind of containers. It is not every time that I had this special card that lists types of investigations and what color of bottle to take the blood in. I would remember some investigations that required a green-top bottle but our laboratory does not have such. One night I was called to review a little boy admitted with dehydration and febrile illness. After clerkship I drew blood samples. I first drew sample for complete blood count in the anticoagulant coated bottle. When filling the plain bottle for electrolytes I realized I could not get enough as blood had already clotted and the child was irritable and restless. I decided to put a little of the sample from the anticoagulant coated bottle and got enough for the plain bottle as well. I was later woken up in the middle of the night by the laboratory people saying the patient had unrecordable hyperkalemia! I later learnt that the anticoagulant in the complete blood count bottle was the cause of the ‘hyperkalemia’. So every other day I would call the laboratory or walk there and make any enquiries that I had in mind, and I realized there were things I should have known but I did not know.

## Implications about our education and human resource aspects of the healthcare system

There is a disturbing unfamiliarity and difference between the teaching hospitals and the rural hospitals where most interns are placed. Such calls for thorough orientation not in terms of ‘where and what’ about the new hospital but in terms of ‘how differently we do it here’. This is the first area of learning brought out in this study. That the most challenging encounters occur when the intern is alone and there are few staff (such as during the weekends and holidays and at night when one is on call) is known to us [2]. This in effect means it is hard for the hospital administration and the seniors to predict all possible disturbing scenarios and develop a ‘manual for orienting interns’. Perhaps a need-based orientation where interns give a feedback about the challenges after every so often and get advice on the way forward would suffice. Such is the prerogative of respective hospitals and the Kenya medical practitioners and dentists board which regulates internship. This would only happen in the first few weeks when the interns are still anxious and least efficient [2].

This paper may seem to portray the nurses as being the custodians of knowledge and the missing link between an intern's deficit and appropriate patient management. But it is the constant critical contact between the critical patient and the nurse that thrusts such importance unto them. Thus, although the author notes above that on a day to day running of a hospital everything rotates around the intern, when narrowed down to every particular patient that focus of centrality shifts from the intern to the nurse. No wonder they know more about certain medications that the patient would require not necessarily because they are more knowledgeable about its pharmacology but because they have the solution that will work in the short term. The intern really has no time to go to the pharmacy and make enquiries when he has a patient who needs urgent intervention. Since they are the ones who administer the medications that the medics prescribe, nurses would be best placed to know what may not be available and what just appears ‘strange’ in a given treatment chart. Most of the nurses may not boast of academic accolades but they definitely possess an undisputed edge over the intern in terms of anecdotal experience (“…I usually see them given these…”).

It is protocol in medicine world over that doctors give instructions and nurses and paramedics implement. So it comes out as a surprise that nurses can disregard a doctor's plan (“… go ahead and prescribe but me I will not give it”)! Probably the juniority and obvious experience deficiency on the intern prompted such a response. It would be exciting to find out how nurses would behave if for example an experienced medical officer or a consultant prescribed an obviously wrong medication. Would they have the guts to ‘just not give it’, and tell that to the doctor? They are however vindicated in that they give the logic behind their assertions (“….he will get hyperglycemia…”). They are not rigid when out-reasoned, and this underscores the importance of team work as emphasized by Brennan et al [2].

Juxtaposed against the findings by Tallentire and colleagues [5], the nurses in this setting would be the main composition in the theme ‘environmental factors’ which significantly influence a junior doctor's decisions.

During the acute care settings and when the intern is on call, the nurses come out as significant determinants of how promptly a patient is reviewed, at times how the patient is managed. They have to assess the seriousness of the case and weight their information when calling the intern. That interns can get mental block when faced with acute, high stakes cases is not new knowledge [2, 4, 5]. Tallentire et al observe a possible delay in making consultations because of fear of reprisals from the seniors and/or the impression that one can manage the case at hand [5]. This paper brings out an additional input from the nurses even when the seniors have been consulted. May be in situations where hospital based management protocols and algorithms are present and visible in all clinical areas (e.g. on the walls at casualty) the degree of such in-put would be minimal. Another aspect that comes out is that of nurses already convinced of what needs to be done and appearing to call the intern just to do what has to be done (the case of midwives in obstetrics with fetal distress).

Two questions arise here: 1) Is this case that of nurses bullying a young inexperienced new intern who has to walk a trodden path?; 2) Do the nurses dictate the management of patients disregarding what the intern has to say? Answers to these questions can only be imagined in the context of the prevailing environment. There are no situations where the nurses recommended or insisted on things out of the ordinary. It is the author's believe that none of such were done to “prove a point” or humiliate the intern.

However, much as there is a lot the nurses contribute to the initial management of acutely ill patients especially during the early days as the intern grows in confidence and efficiency, a certain worry crops up. How up to date are our nurses when it comes to the latest developments in management of various acute conditions? How willing are they to listen to a new intern who may not demonstrate full confidence but is right in choosing a certain management plan however unfamiliar to the nurses? Well, it all depends on the intern now. One has to be sensitive to such and firm in his decisions, calling the second or third on call if need be to support his stand. As a doctor, the intern ‘must be capable of taking ultimate responsibility for difficulty decisions in situations of clinical complexity and uncertainty’ [2]. Some of these occurrences are firsts for the interns and they will definitely learn from their experiences [2] and handle future cases better. There are a few interns who will confess to their undergraduate medical schools having prepared them well for internship [2, 10]. Goldacre and colleagues observed a variation between different medical schools in UK in terms of how well their graduates felt prepared and recommended systematic in-depth feedbacks to medical schools from their alumni [10]. A few years later, their recommendations were supported by Greenburg and colleagues who concluded that it was difficult to predict the likely professionalism and performance of interns by gauging them using medical school performance [8].

It is worth to advise on medical curriculum modifications where “meaningful contact” [2] with the patient takes place in terms of giving senior medical students the chance to work up and prescribe treatments, follow up the patients etc. Tallentire et al [5] attempted to understand the behavior of newly qualified doctors in acute care settings while Brennan and colleagues [2] studied the experiences of newly qualified doctors in the entire hospital setting. Besides Brennan et al [2] finding out that surgeons were so busy they did not have time to mentor and give direction to the young doctors, the researchers did not find out the aspects of lack (or mastery) of surgical skills among the new graduates. Studies assessing surgical skills competence are mostly directed towards surgical residents. It would be necessary to know how undergraduates fare in terms of mastery of simple surgical skills and instruments. In the author's setting, theater assistants and scrub nurses come out as playing a role in mitigating this deficit. Though surgery is more of apprenticeship, a little more of this apprenticeship at the undergraduate level can help a great deal for the interns bound for rural Kenya.

Is knowledge of sutures and instruments used in theater an approximate representation of “meaningful exposure” [2] to surgical patients in undergraduate training?

Apart from theater, it appears there is little the other paramedics offer the intern. Only the laboratory technicians come out as playing a role here. In centers that have phlebotomists, the interaction between the intern and the laboratory technician would definitely be diminished.

We need studies that look into the interactions between the nurse and the intern. That way we can understand if there is influence of inexperience in how nurses interact with the interns. There is also need for frequent feedback to medical schools by their alumni in terms of what can be improved on in the fields of clinical exposure for undergraduate students. Finally a study that assesses the surgical skills gained in the medical school would highlight if surgery is the neglected department when it comes to practical exposure for undergraduate medical students.

## Conclusion

Internship is a learning period. Irrespective of what competence interns come with into the field there is still a lot they have to learn from the nurses and the paramedics. Most of the learning takes place during the acute care settings when the intern is on call and is from the nurses. The paramedics most helpful to the intern are the theater assistants who teach interns how to use the various surgical instruments and sometimes direct during minor operations.
